# A Hierarchical Method for Removal of Baseline Drift from Biomedical Signals: Application in ECG Analysis

**DOI:** 10.1155/2013/896056

**Published:** 2013-05-20

**Authors:** Yurong Luo, Rosalyn H. Hargraves, Ashwin Belle, Ou Bai, Xuguang Qi, Kevin R. Ward, Michael Paul Pfaffenberger, Kayvan Najarian

**Affiliations:** ^1^Department of Computer Science, School of Engineering, Virginia Commonwealth University, 401 West Main Street, Richmond, VA 23284, USA; ^2^Department of Electrical and Computer Engineering, School of Engineering, Virginia Commonwealth University, 401 West Main Street, Richmond, VA 23284, USA; ^3^Department of Biomedical Engineering, School of Engineering, Virginia Commonwealth University, 401 West Main Street, Richmond, VA 23284, USA; ^4^Department of Emergency Medicine and Michigan Critical Injury and Illness Research Center, University of Michigan, Ann Arbor, MI 48109, USA

## Abstract

Noise can compromise the extraction of some fundamental and important features from biomedical signals and hence prohibit accurate analysis of these signals. Baseline wander in electrocardiogram (ECG) signals is one such example, which can be caused by factors such as respiration, variations in electrode impedance, and excessive body movements. Unless baseline wander is effectively removed, the accuracy of any feature extracted from the ECG, such as timing and duration of the ST-segment, is compromised. This paper approaches this filtering task from a novel standpoint by assuming that the ECG baseline wander comes from an independent and unknown source. The technique utilizes a hierarchical method including a blind source separation (BSS) step, in particular independent component analysis, to eliminate the effect of the baseline wander. We examine the specifics of the components causing the baseline wander and the factors that affect the separation process. Experimental results reveal the superiority of the proposed algorithm in removing the baseline wander.

## 1. Introduction

The electrocardiogram (ECG) is an important physiological signal that helps determine the state of the cardiovascular system; however, this signal is often corrupted by interfering noise. Baseline wander is a commonly seen noise in ECG recordings and can be caused by respiration, changes in electrode impedance, and motion. Baseline wander can mask important information from the ECG, and if it is not properly removed, crucial diagnostic information contained in the ECG will be lost or corrupted. Therefore, it is vital to effectively eliminate baseline wander before any further processing of ECG such as feature extraction.

The simplest method of baseline wander (drift) removal is the use of a high-pass filter that blocks the drift and passes all main components of ECG though the filter. The main components of ECG include the P-wave, QRS-complex, and T-wave. Specifically, the PR-Segment, ST-Segment, PR-Interval, and QT-Interval are considered as the main segments of the ECG. Each of these intervals/segments has its corresponding frequency components, and according to the American Health Association (AHA), the lowest frequency component in the ECG signal is at about 0.05 Hz [1]. However, a complete baseline removal requires that the cut-off frequency of the high-pass filter be set higher than the lowest frequency in the ECG; otherwise some of the baseline drift will pass through the filter. The frequency of the baseline wander high-pass filter is usually set slightly below 0.5 Hz. Therefore, knowing that the actual ECG signal has components between 0.05 Hz and 0.5 Hz, the forementioned simple approach for baseline removal distorts and deforms the ECG signal. In particular, it affects the ST-segment that has very low frequency components. Furthermore, ectopic beats occurring in the ECG during the course of different types of diseases and injuries change the frequency spectrum of both the baseline wander and the ECG waveforms. All the above-mentioned characteristics demand a more comprehensive approach that works for a wider range of applications and avoids distorting the main ECG waves when removing the baseline drift.

Digital filters are commonly employed method to eliminate baseline wander. Cut-off frequency and phase response characteristics are two main factors considered in the majority of these designs. The use of linear phase filters prevents the issue of phase distortion [2]. For finite impulse response (FIR) filters, it is rather straightforward to achieve linear phase response directly. Feed-forward and feed-back technologies such as infinite impulse response (IIR) filters can also provide minimum phase distortion [3]. In all of these methods, the cut-off frequency should be chosen so that the information in the ECG signals remains undistorted while the baseline wander is removed, which results in a trade-off. Usually, the cut-off frequency is set according to the slowest detected (or assumed) heart rate. However, if there are ectopic beats in the ECG signal, it is even more difficult to find this particular frequency. It is a prevalent phenomenon that the overlap between the baseline wander and low frequency components of the ECG compromises the accuracy of the extracted features. 

Time-variant filters are designed to increase flexibility in the adjustment and control of the cut-off frequency. In such methods, the cut-off frequency of the filter is controlled by the low frequency characteristics of the ECG signal [4]. Cubic spline curve fitting [5], linear spline curve fitting [6], and nonlinear spline curve fitting [7] belong to another family of filters that remove the baseline wander but often require some reference points. For instance, the linear spline curve fitting method [5] forms a subsignal of the ECG for a single cardiac cycle starting 60 ms before the P-wave and ending 60 ms after the T-wave and fits a first order polynomial to this sub-signal after subtracting the mean of sub-signal. Multirate system wavelet transform has also been utilized for the ECG baseline wander removal. The approach using wavelet adaptive filter (WAF) [8] consists of two steps. First, a wavelet transform decomposes the ECG signal into seven frequency bands. The second step is an adaptive filter that uses the signal of the seventh lowest frequency band as the primary input and a constant as a reference unit for filtering. Another multi-rate system, empirical mode decomposition (EMD) [9], has also been adopted to eliminate the baseline wander. Compared with the wavelet technique that uses some predefined basis functions to represent a signal, EMD relies on a fully data-driven mechanism; that is, EMD does not require any a-priori known basis.

Adaptive filters as a cascade structure [10] have also been used for this application. The first step of this approach uses an adaptive notch filter to eliminate the DC component of the ECG. The second step forms a comb filter assuming that the signal is an event-related signal. Blind source separation (BSS), in particular independent component analysis (ICA) [11–13], is another choice to remove the baseline wander. As a specific type of BSS method, ICA has been extensively used in biomedical signals [14–16], such as the ECG and the EEG. It has been used as an effective method to decompose multichannel signals into fundamental components. As many more applications of ICA are being recognized, newer variations of ICA are being introduced. Standard ICA [17] (sICA) is a technique that is used to estimate source signals when several mixtures of signals are available. Both the source signals and the mixing process are unknown, and the sources are estimated only on the assumption that they are statistically independent. Comparing the formulation of the standard ICA, convolutive ICA (fICA) deems that the finite impulse response is closely associated with the mixing process, and the mixing process can be considered as a weighted and delayed mixture of sources [18, 19]. Fast and robust fixed-point ICA [20] is produced based on the idea that it is feasible to use contrast function to approximate negentropy. Through a fixed-point algorithm, the contrast function is maximized to extract latent sources with high speed. Temporally constrained ICA [21, 22] is a more flexible model to separate latent sources. By using prior knowledge or additional constraints, the targeted latent source is extracted. Moreover, there are many other forms of ICA for different applications such as topographic independent component analysis [23] and spatial and temporal independent component analysis [24].

In summary, the traditional methods are limited in either frequency delineation or reference choice, and the case of BBS in applications mentioned previously does not give sufficient evidence in noise removal. Based on these points of view, in the proposed method, a unified method utilizing an adaptive notch filter and BSS is used for baseline drift removal. Specifically, multichannel signals are constructed using a single-channel signal, and ICA is applied to the ECG. The main contributions of our work lie in combining the capabilities of adaptive filters and BSS, expanding the capabilities of the independent components for this application by customizing the ICA method towards the removal of the ECG baseline wander. Furthermore, the factors affecting the performance of the separation process are explored and improved in this paper.

The rest of the paper is organized as follows. The overall structure of the proposed method is illustrated in Section 2. The adaptive notch filter, as employed in the paper, is described in Section 3. The concepts and formulation of the ICA, the fast and robust fixed-point ICA, and the customized form are introduced in Section 4.  Section 5 introduces the process of detecting the components that cause the baseline wander and verifies this process. This section also explores the factors that affect the separation of the results. Finally, Section 6 concludes the paper.

## 2. Method

**Figure 1:**
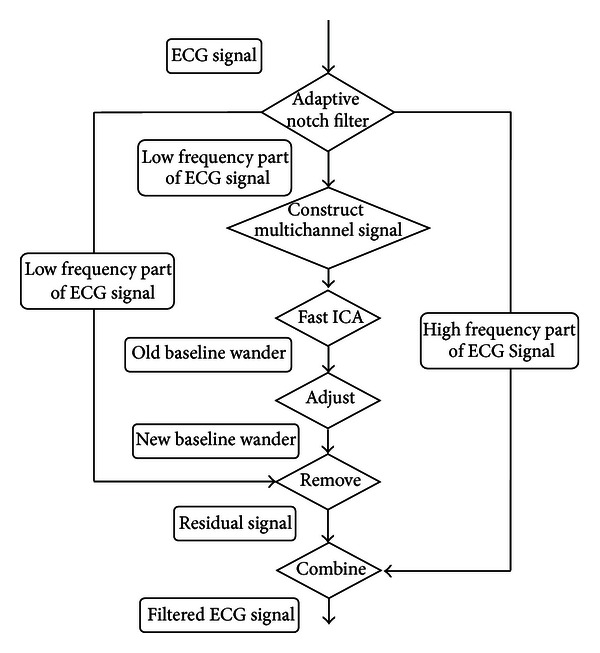
Schematic diagram of proposed method.

Figure 1 shows the framework of the proposed method. As it can be seen in Figure 1, the first step of the proposed method is an adaptive notch filter, designed to form subsignals of the ECG, as described later. Next, as shown in Figure 1, the proposed method utilizes ICA to remove the baseline drift. Considering the noisy nature of the typical raw ECG signal, in this study, subsignals in low frequencies of the ECG are formed and these filtered signals are, then, formed by an adaptive notch filter, then used as the input to the ICA algorithm. Moreover, with regard to the inputs fed to the ICA algorithm, in this study, only a single-channel ECG signal is available. Therefore, knowing that ICA requires multichannel signals to process as its input, in order to use ICA to remove baseline wander, one needs to build multichannel signals from the single-channel ECG. In order to address this issue in the proposed method, a systematic process was created in which delayed versions of the ECG are stacked to form the multi-channel signal. In addition, as shown in Figure 1, the independent component formed by the ICA as the output, which is originally labeled as the baseline wander, needs to be further adjusted to form a better estimate of the baseline wander. This is due to the fact that, while one of the components resembles the baseline drift, it is unlikely that any of the original components detected by the ICA is “purely” the baseline wander.

The specific steps shown in Figure 1 are further described below.

- Form sub-signals of ECG using an adaptive notch filter: as shown in Figure 1, the adaptive notch filter [25, 26] is designed and customized to form the sub-signal. The reason for using the adaptive notch filter is its flexibility as well as its relatively superior performance compared with other filters. As mentioned above, applying the ICA algorithm on a sub-signal of the ECG has the advantage of reducing the errors coming from multi-channel signals in estimating the baseline wander.
- Construct multi-channel signals: applying ICA requires that the signals are multi-channel ones. However, in many ECG processing applications only the single-channel ECG signal is available and/or processed. The proposed method applies the methodology in [11] to construct multi-channel signals by delaying the single-channel signal. In our study, the multi-channel signals are constructed using sixty signals, which are delayed 10 sample points (~83 ms) of the original signal in succession.
- Adjust the baseline wander extracted by ICA: the baseline wander extracted by ICA is an approximation of the true baseline wander because (1) there will be some errors in the resulting component due to the fact that the estimation process used in the ICA (in particular in the first few attempts) may be nonoptimal; (2) in the ICA analysis there may be more than one maximum in the estimation function and, therefore, the true baseline wander may not be located accurately; (3) the constructed multi-channel signals cannot convey all information about the baseline wander and, as such, the proposed process may alleviate the issues associated with the non-optimal construction of multi-channel signals. The 10-sample shift of the signals provides large enough variations between the multisignal component to alleviate the issues concerning dependencies for ICA processing.

## 3. Adaptive Notch Filter

The adaptive notch filter [26] is based on the same theoretical foundations as adaptive noise cancellation [25]. There are two inputs in the structure of the adaptive noise cancelling. One is the primary input, containing the signal and the noise and the other one is the reference input, which is the reference signal related to the noise in the primary input. Using least mean square (LMS) criterion, the reference signal is gradually approached to the noise in the primary input. When the stability is achieved, the output is acquired through subtracting the reference input from the primary input. This type of filter can deal with inputs that are deterministic or stochastic, stationary or time-variant. If the inputs are stationary stochastic, the solution of the adaptive noise cancelling approaches closely Wiener filter [25]. As to the adaptive notch filter, the reference signal is the signal with one- or multifixed frequencies, which are treated as the frequencies to be excluded.

**Figure 2:**
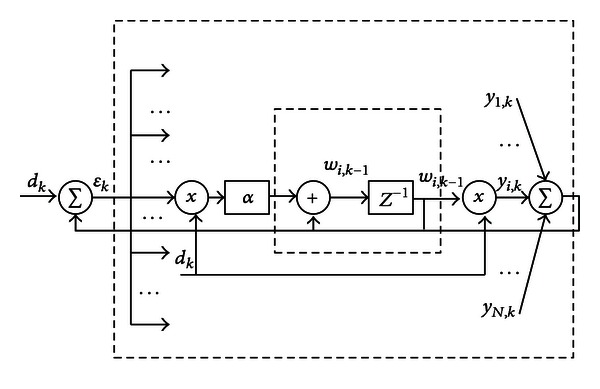
The diagram of adaptive noise cancelling.

The advantages of adaptive notch filters lie in the following aspects: (1) if the frequency of the interference is not precisely known or the interference drifts in the frequency, the exact excluded frequency could be measured/adapted during the filtering process; (2) the filter is tunable since the null point moves with the reference frequencies; (3) the adaptive notch filter can be made very sharp at the reference frequency; (4) through adjusting the parameters, the adaptive notch filter can be considered as a time-invariant filter by lessening the influence of the time-varying components. The inference of adaptive notch filter is described in [25, 26]. The diagram of adaptive noise cancelling is shown in Figure 2. The system is an *N*-stage tapped delay line (TDL). The weight of the filter is updated according to the following equations:

(1) $$\begin{array}{c} y_{k}=w_{k}^{T}x_{k},\\ \varepsilon_{k}=d_{k}-y_{k},\\ w_{k+1}=w_{k}+\partial \varepsilon_{k}x_{k}, \end{array}$$

where *x* is the reference input, *d* is the desired response, *y* is the output of the filter, *w* is the weight of the filter, ∂ is the adaptation constant, and *k* is the time index. As described in [26], the response from *E*(*z*) to *Y*(*z*) includes two parts. In practical applications, it is feasible to make the time-varying component to be insignificant (*β*/*N* ≈ 0) by changing the values of *N* and setting *β* as follows:

(2) $$\begin{array}{c} \beta =\frac{\sin\left(Nw_{r}T\right)}{\sin\left(w_{r}T\right)}, \end{array}$$

where *w*_*r*_ is the frequency of the interference. If the reference input is considered to be the following form:

(3) $$\begin{array}{c} x=C\cos\left(w_{r}T+\theta \right), \end{array}$$

the transfer function of adaptive notch filter can be expressed as follows:

(4) $$\begin{array}{c} H\left(z\right)=\frac{z^{2}-2z\cos\left(w_{r}T\right)+1}{z^{2}-2\left(1-N\partial C^{2}/4\right)z\cos\left(w_{r}T\right)+\left(1-N\partial C^{2}/2\right)}. \end{array}$$

Therefore, the parameter *N* can be set to the fixed value as described above. It can be seen that the above-mentioned filter is very flexible and can be adjusted using the adaption constants ∂ and *C* to provide the desired bandwidth and depth of a suitable notch filter.

## 4. Independent Component Analysis

After applying the notch filter, the main step used is ICA. First, the “standard” ICA is described. ICA can be briefly explained using a simple example of separating two source signals *s*_1_(*t*) and *s*_2_(*t*) that were mixed by an unknown linear process. Two different linear mixtures, *x*_1_(*t*) and *x*_2_(*t*), are given as follows:

(5) $$\begin{array}{c} x_{1}\left(t\right)=c_{11}s_{1}+c_{12}s_{2},\\ x_{2}\left(t\right)=c_{21}s_{1}+c_{22}s_{2}, \end{array}$$

where *c*_11_, *c*_12_, *c*_21_, and *c*_22_ are unknown coefficients. The objective of the problem is to recover the signal *s*_1_(*t*) and *s*_2_(*t*) from mixture signals *x*_1_(*t*) and *x*_2_(*t*) without knowing any prior information about the source signals *s*_1_(*t*) and *s*_2_(*t*) and the mixing process (i.e., *c*_11_, *c*_12_, *c*_21_, and *c*_22_), except that *s*_1_(*t*) and *s*_2_(*t*) are statistically independent. 

In the generalized case, where there are more latent sources and more mixture of signals, the formal definition of ICA is as follows:

(6) $$\begin{array}{c} x_{i}\left(t\right)=c_{i1}s_{1}+c_{i2}s_{2}+\cdots +c_{in}s_{n},\;i\in \left[1,n\right], \end{array}$$

where *s*_*i*_(*t*) is called latent source, *x*_*i*_(*t*) is the mixture signal, *c*_*ij*_ is the mixing coefficient between *x*_*i*_(*t*) and *s*_*j*_(*t*), and *n* is the number of latent sources and mixture signals. The above formulation can be expressed as the following matrix form:

(7) $$\begin{array}{c} X=C_{n\times n}\cdot S, \end{array}$$

where *X* is the matrix of mixture signals, in which each column is one mixture signal; *S* is the matrix of latent signals, in which each column is one latent signal; and *C*_*n*×*n*_ is the matrix for mixing coefficients.

The feasibility of solving the ICA problem lies in the condition that the latent sources are independent of each other. According to the Central Limit Theorem, the distribution of a sum of independent random variables approaches a Gaussian distribution. This implies that the solution of ICA can be achieved when distribution diverges from Gaussianity. The deviation from Gaussianity can be determined using measures such as Negentropy.

Negentropy is one measure of non-gaussianity defined based on the concept of entropy, which is the fundamental concept of information theory. Entropy, *E*, as a measure of information in random variables is defined for a discrete random variable *y* as folows:

(8) $$\begin{array}{c} E\left(y\right)=-\sum_{i}P\left(y=c_{i}\right)\log P\left(y=c_{i}\right), \end{array}$$

where *c*_*i*_ is the possible values of *Y* and *P*(*Y* = *c*_*i*_) means the probability when the value of *Y* is *c*_*i*_. For a continuous random variable *y*, entropy *E* is defined as the following equation:

(9) $$\begin{array}{c} E\left(y\right)=-\int f\left(y\right)\log\left(f\left(y\right)\right)dy, \end{array}$$

where *f* is the probability distribution function. Negentropy, *J*, is then defined as follows:

(10) $$\begin{array}{c} J\left(y\right)=E\left(y_{\text{gauss}}\right)-E\left(y\right), \end{array}$$

where *y*_gauss_ is a Gaussian random variable with the same covariance matrix as *y*. A fundamental conclusion in information theory is that a Gaussian variable has the largest entropy among all random variables of equal variance. Hence, negentropy is always nonnegative, and it is zero only if *Y* has a Gaussian distribution.

The exact calculation of negentropy requires an accurate estimation of the probability distribution function, which may be computationally costly or data intensive. Therefore, it is often preferred to find simple approximations of negentropy. Simple approximations of negentropy have been introduced [27], which are based on the maximum entropy principle. In general, the following family of approximations is the most commonly used group:

(11) $$\begin{array}{c} J\left(y\right)=\sum_{i=1}^{p}k_{i}{\left[E\left(G_{i}\left(y\right)\right)-E\left(G\left(v\right)\right)\right]}^{2}, \end{array}$$

where *k*_*i*_ are constants and *v* is a gaussian random variable with zero mean and unit variance. Often, the value of *p* and *k*_*i*_ can be set to one. Therefore, the above formulation becomes as follows:

(12) $$\begin{array}{c} J\left(y\right)={\left[E\left(G\left(y\right)\right)-E\left(G\left(v\right)\right)\right]}^{2}. \end{array}$$

The following formulations of *G* functions have proved very useful in practical applications:

(13) $$\begin{array}{c} G_{1}\left(y\right)=\frac{1}{a_{1}}\text{log cosh}\left(a_{1}y\right),\;\;g_{1}\left(y\right)=\tanh\left(a_{1}y\right),\\ G_{2}\left(y\right)=-\frac{1}{a_{2}}\exp\left(-\frac{a_{2}y^{2}}{2}\right),\;\;g_{2}\left(y\right)=y\exp\left(-\frac{a_{2}y^{2}}{2}\right),\\ G_{3}\left(y\right)=\frac{1}{4}y^{4},\;\;g_{3}\left(y\right)=y^{3}, \end{array}$$

where 1 ≤ *a*_1_ ≤ 2, *a*_2_ ≈ 1, and *g* is the first derivative of the function *G*.

Before applying the main processing operations of the ICA, it is often necessary to perform some preprocessing. Usually, the two different operations are conducted: centering and whitening. Centering requires that the random variable *y* is a zero-mean random variable, and it is performed by subtracting its mean vector. Whitening will make the random variable uncorrelated and set their variances equal to unity by using the eigenvalue decomposition of their covariance matrix:

(14) $$\begin{array}{c} E\left\{yy^{T}\right\}=DVD^{T}, \end{array}$$

where *D* is the orthogonal matrix of eigenvectors and *V* is the diagonal matrix of eigenvalues. Now, assuming that *z* is a new random variable after whitening, consider the following:

(15) $$\begin{array}{c} z=DV^{-1/2}D^{T}y. \end{array}$$

Whitening makes the problem change from estimating mixing matrix to estimating a new one $\tilde{C}$:

(16) $$\begin{array}{c} z=DV^{1/2}D^{T}Cs=\tilde{C}s. \end{array}$$

Among several improvements of ICA, fast and fixed-point independent component analysis [20], as a direct extension of the standard ICA, was developed for calculating latent sources with high speed. The basic rule of fast and fixed-point independent component analysis is to find a direction, which can maximize non-Gaussianity of *w* ^*T*^ *x*. Non-Gaussianity is decided according to the approximation of nongaussianity as mentioned above. The following is the basic description of the algorithm.

- Initialize a weight vector *w* in one direction.
- Change the weight vector according to the following criteria: *w*′ = *E*{*xg*(*w* ^*T*^ *x*)} − *E*{*g*′(*w* ^*T*^ *x*)}*w*, and normalize the weight vector as *w* = *w*′/||*w*′||.
- If the weights have not converged, go back to step (b),

where *w* is the weight vector to calculate latent source *s* = *w* ^*T*^ *x* and convergence means that the old weight vector and the new weight vector are in the same direction.

In this study, the fast and fixed-point independent component analysis [20] is used as the implementation of ICA block shown in Figure 1.

## 5. Results

An ECG dataset of human volunteer undergoing lower body negative pressure (LBNP) [28] as a surrogate of hemorrhage was employed to verify the effectiveness of removing baseline wander. This data set was created under Institutional Review Board approval. The LBNP dataset consisted of a total of 91 subjects. Each subject had a single vector lead ECG recording collected at the sampling rate of 500 Hz. The baseline wander in ECG signals demonstrated significant level of variations in the amplitude over the course of the LBNP experiment. During LBNP, subjects are exposed to increasing negative pressure to their lower bodies. This causes a redistribution of blood volume to the lower extremities and abdomen causing a decrease in blood pressure and cardiac output and resulting in an increased respiratory rate.

The results of the proposed method are compared with a reference method, called robust locally weighted regression [29], which is often treated as one of the most robust and commonly used methods to remove baseline drift. The robust locally weighted regression method employs two techniques: the local fitting of polynomials and an adaptation of iterated weighted least squares to remove the baseline drift.

### 5.1. Results of Adaptive Notch Filter

**Figure 3:**
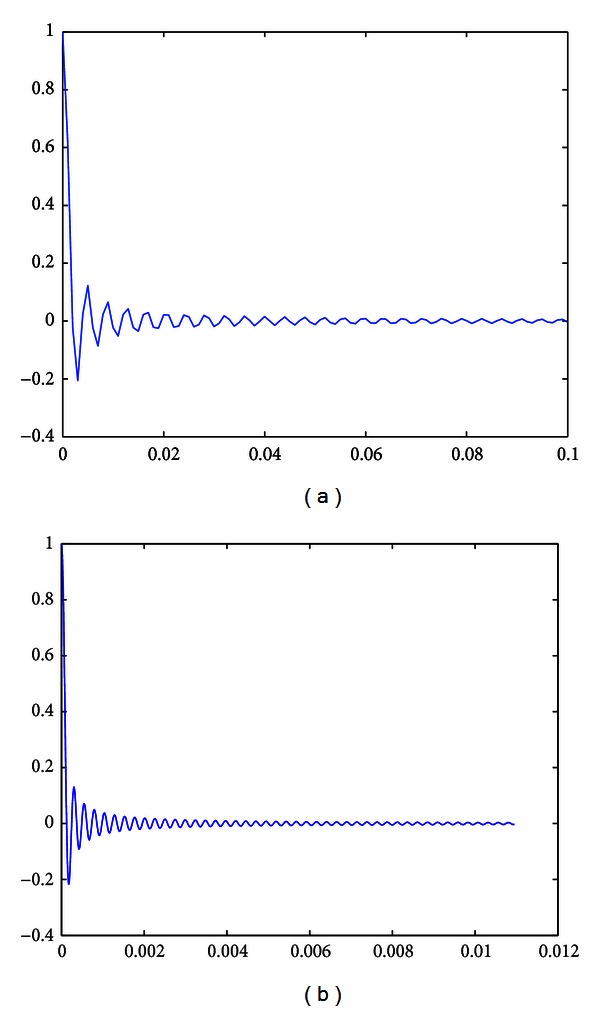
The resulting value of *β*/*N* (a) *N* = 256; (b) *N* = 4096.

One objective of the proposed system is the removal of unwanted frequencies around 0 Hz as well as 60 Hz. As the frequencies around zero are excluded, the filter acts as a high-pass filter. In order to lessen the influence of the time-varying components, one needs to first set a suitable parameter *N* to obtain a desirable level of time-varying component, *β*/*N*.  Figure 3 shows the value of the time-varying component *β*/*N* for different values of *N*.

Figure 3 indicates that the value of *N* determines the degree at which the time varying component influences the filter. In general, with the increase in the value of *N*, this influence decreases gradually. In this study, the value of *N* was set to 10,000. The parameter ∂ identifies whether or not the adaptation converges [25]. The value of ∂ should be greater than 0 but less than the reciprocal of the largest eigenvalue, *λ*, of the matrix *R*, which is defined as the correlation matrix of signal [25]. In this study, the value of ∂ was set to 0.0001. The bandwidth of the filter can be approximated using the following equation [26]:

(17) $$\begin{array}{c} \text{BW}=\frac{N\partial C^{2}}{2T}\;\;\left(\text{rad}/\text{s}\right). \end{array}$$

**Figure 4:**
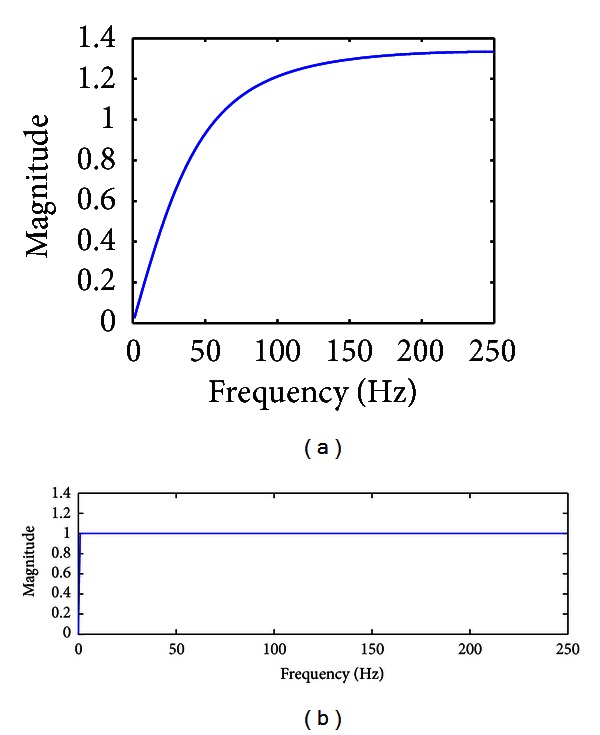
Transfer function for two choices of adaptive notch filters (a) *C* = 1; (b) *C* = 0.01.

Figure 4 shows the transfer function of the resulting adaptive notch filter, and, as expected, this filter acts as a high-pass filter. Note that the value of *C* provides yet another degree of freedom for this filter design process, and, hence, Figure 4 presents the transfer function for two different filters formed using two different values of *C*, each resulting in a very different bandwidth. A main advantage of the adaptive notch filter used here is that changing the values of parameters *N*, ∂, and *C* can provide a wide spectrum of desired filters with diverse shapes of transfer function.

**Figure 5:**
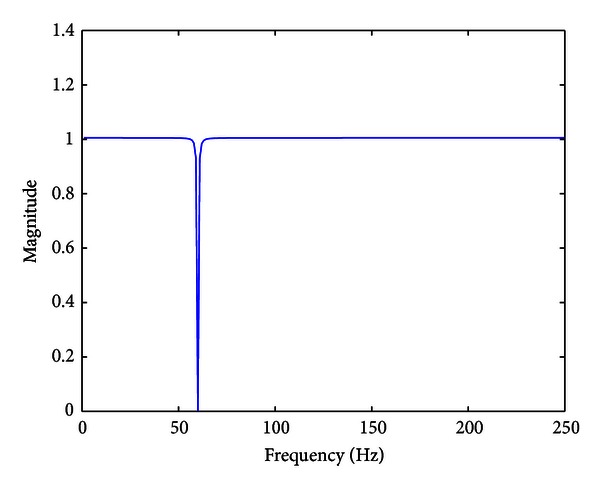
Transfer function of the adaptive notch filter around 60 Hz.

Adaptive notch filter for frequencies around 60 Hz is designed similarly. The parameter *N* was to 2048, ∂ to 0.001, and *C* to 0.1.  Figure 5 depicts the transfer function of the resulting adaptive notch filter.

### 5.2. Experimental Results and Problems Analysis

The results of both methods, that is, the proposed and the reference methods, are examined and compared in all 91 subjects. A unified “span” value, described in the reference method [29], which is designed to assess the quality of the methods in removing the baseline wander, is calculated for all cases. This value for all experimental results was 1500, which is the level identifying a very high quality of baseline removal. 

The 91 cases, based on the closeness of the results of the two methods, are divided into two groups. The details of the results are shown for 72 out of 91 subjects in Table 1; for these subjects the proposed algorithm achieves almost identical results as the reference method. The results of the remaining 19 subjects, which will be discussed separately, show that the proposed method cannot be able to remove the baseline drift optimally.

In Table 1, “shift” and “elevation” are the values for adjustments to the original independent component (baseline wander) to form the new baseline wander in the horizontal and vertical directions; “error_1_” represents the difference between the old baseline wander (sig_1_) before shift and the baseline wander (sig) from the reference method calculated as follows:

(18) $$\begin{array}{c} {\text{error}}_{1}=\frac{{\left({\text{sig}}_{1}-\text{sig}\right)}^{2}}{n}, \end{array}$$

where *n* is the number of sample points in the baseline wander, and finally “error_2_” represents the difference between the new baseline wander (sig_2_) and the baseline wander (sig) from the reference method calculated as follows:

(19) $$\begin{array}{c} {\text{error}}_{2}=\frac{{\left({\text{sig}}_{2}-\text{sig}\right)}^{2}}{n}. \end{array}$$

As it can be seen in Table 1, for all cases error_2_ is significantly smaller than error_1_ which shows the impact of that method in “purifying” the baseline wander and creating a better estimate of the drift. In order to better assess the performance of the proposed method in removing the baseline wander, more analyses are conducted on the results.

**Figure 6:**
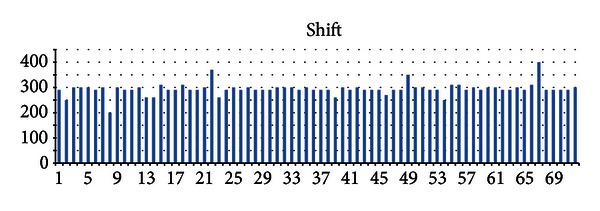
Value of “shift” that adjusts the old baseline wander to form the new one for all 72 subjects.

**Figure 7:**
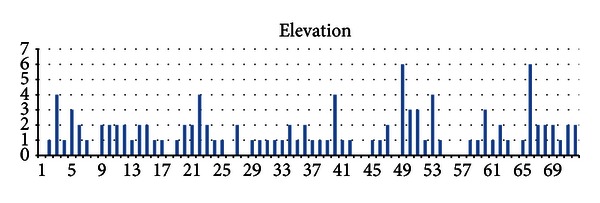
Value of “elevation” that adjust the old baseline wander to form the new one for all 72 subjects.

Figures 6 and 7 show the shift and elevation for all 72 subjects. As can be seen, both of these variables are almost the same for all subjects and do not change across different subjects (*x*-axis) or vary in a small scope. This observation illustrates the reason to adjust the parameters between the old baseline wander and the new baseline wander.

**Figure 8:**
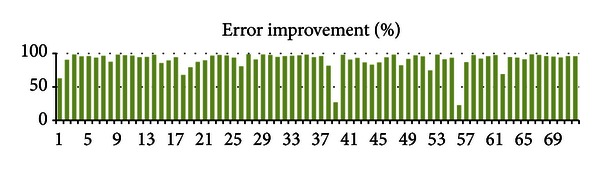
Improved percentages of error after adjustment.

Figure 8 shows the error reduction in 72 subjects after adjusting shift and elevation value. It can be seen that in all of these cases the errors decrease significantly after adjusting the baseline wander compared with the baseline wander. The average percentage of error reduction Aver *E* reaches up to 90.13%. The formulation of the average percentage of error reduction is shown in the following:

(20) $$\begin{array}{c} \text{percentage}\left(i\right)=\frac{\left({\text{error}}_{1}-{\text{error}}_{2}\right)}{{\text{error}}_{1}},\;i\in \left[1,n\right],\\ \text{Aver}\;E=\sum_{i=1}^{i=n}\text{percentage}\left(i\right), \end{array}$$

where *i* is the index of subject and *n* is the total number of subjects.

**Figure 9:**
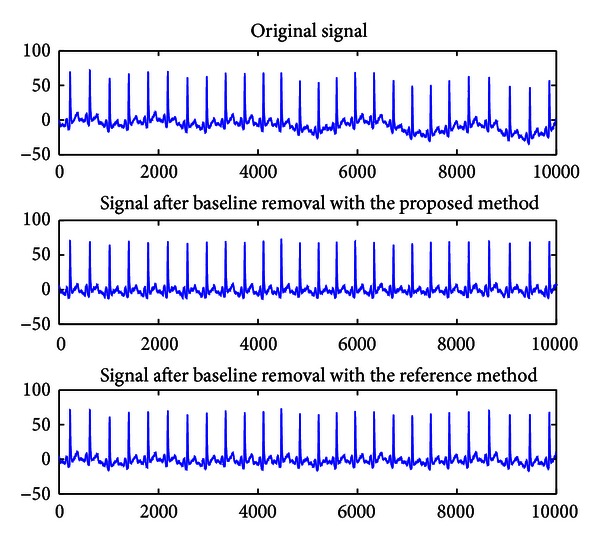
Comparison between the proposed method and the reference method.

Sample signals before baseline removal and after baseline removal with the proposed method as well as the reference method are shown in Figure 9. As shown in Figure 9, the results of the two methods in all above-mentioned 72 subjects are very similar. In addition, as it can be seen, both methods are very effective in removing the baseline drift.

However, as mentioned above, on the ECG of the remaining 19 subjects, the results of the proposed method and the reference method are not as similar; that is, the value of error_2_ (which shows the difference between the two methods) is significant. This is because in these signals the inherent pattern observed from ECG is highly distorted hence leading to spurious estimations. As mentioned before, we have visually inspected all 91 cases. By examining the signals for these 19 cases, it was discovered that the high value of error_2_ does not seem to come from the inability of the proposed method to remove the baseline wander. In such case, the possible reason and improvement are discussed in the following part.

**Figure 10:**
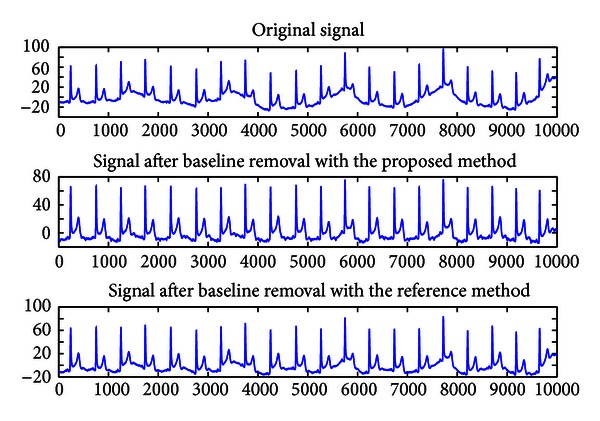
Comparison of the proposed method to the reference method.

As a comparison between the proposed method and reference method, some such sample results are shown in Figure 10. In these cases, due to the presence of significantly stronger baseline drifts, the reference method seems not to be eliminating almost all the baseline drift. The reason for this might lie in the fact that the reference method relies heavily on the parameters set that may work very well for some ECG signals but not for others. As shown in Figure 10, our proposed method shows more effective performance in removing the baseline around times such as 4700, 5500, 7500, and 9500. Another major advantage is that the proposed method is computationally faster than the reference method while achieving the same quality of results. 

### 5.3. Further Experimental Analysis of Method

As mentioned above, in the experiment, multi-channel signals are constructed through a single-channel signal. The multi-channel signals are constructed using sixty signals, which are 10 sample point delayed successions of the original signal. By observation, the number of the constructed signals greatly impacts the success of finding the true baseline wander. Moreover, the degree of delay has a close relationship with the smoothness of the baseline wander. Experimentally, it can be considered that more channels and smaller delayed signals may achieve better results, meaning that the constructed multi-channel signals may convey enough information in order to accurately extract the baseline wander.

In addition, as discussed above, the LBNP dataset shows a significant level of variations in the baseline drift. Therefore, in further analysis of the method, the sub-signals were segmented to verify whether the slow changes in the trend of the baseline wander affect the results of the proposed method in separating the baseline wander. The sub-signals were chosen to be only 10,000 sample points long from the beginning of the original signal in LBNP dataset. Experimental results showed that the slow changing trend of the baseline wander did not affect the performance of the proposed method in extracting the baseline wander. In other words, the baseline drift with slow changing trends can also be successfully extracted using the proposed method.

## 6. Conclusion

While using the blind source separation paradigm, the ECG baseline wander or drift may be removed. The present paper demonstrates a hierarchical method utilizing ICA to significantly improve the performance of this process and achieve improved performance. Compared with the existing methods, the proposed method has the following advantages. (1) The proposed method provides more flexibility with regard to parameter estimation and selection. (2) When following the steps proposed for adjustment of ICA process, the fundamental assumption of baseline noise coming from an independent source can be further verified, which supports the validity of using the method for ECG baseline removal. Such an assumption, verified by additional experimental results, would present a chance to remove other types of noise. (3) The filtering process proposed for forming the multi-channel signals provides a highly flexible method to form the input to ICA.

## Figures and Tables

**Table 1:** Experimental results of removing the baseline wander.

Subject	Shift/elevation	Error_1_	Error_2_
1	290/0	2.0996	0.7847
2	250/1	28.1832	2.7037
3	300/4	193.9524	3.4495
4	300/1	24.3905	1.0727
5	300/3	89.1358	3.6282
6	290/2	17.9017	1.1614
7	300/1	28.955	1.0623
8	200/0	107.7542	13.4439
9	300/2	203.8138	4.0846
10	290/2	81.7942	2.2818
11	290/2	256.3747	8.7264
12	300/2	41.0977	2.4223
13	260/1	44.2238	2.279
14	260/2	101.7592	2.3317
15	310/2	700.1481	101.429
16	290/1	12.7575	1.3522
17	290/1	45.6429	2.6412
18	310/0	36.8833	11.8224
19	290/1	9.1224	1.88
20	290/2	181.3923	23.0193
21	300/2	25.4492	2.6421
22	370/4	252.4353	8.5616
23	260/2	304.7066	7.4637
24	290/1	116.9048	3.77
25	300/1	16.3922	1.05
26	290/0	3.4748	0.6671
27	300/2	144.4347	1.8579
28	290/0	23.9724	2.1641
29	290/1	14.5089	0.2205
30	290/1	155.3859	3.6707
31	300/1	50.6959	2.6757
32	300/1	27.2665	1.101
33	300/1	56.7045	1.999
34	290/2	324.4399	10.0313
35	300/1	42.6266	0.8791
36	290/2	539.7357	31.3238
37	290/1	19.8874	0.8131
38	290/1	14.3623	2.6499
39	260/1	8.8582	6.4787
40	300/4	135.0286	3.0056
41	290/1	29.5551	2.7541
42	300/1	43.3923	3.0052
43	290/0	51.0465	6.9241
44	290/0	31.9213	5.4646
45	290/1	9.7597	1.3328
46	270/1	22.7897	1.3598
47	290/2	93.5265	1.899
48	290/0	8.7422	1.5607
49	350/6	892.829	74.3034
50	300/3	209.4986	6.3436
51	300/3	60.5121	2.6645
52	290/1	3.7123	0.9486
53	290/4	247.2271	5.138
54	250/1	32.0128	2.8609
55	310/0	20.1471	1.336
56	310/0	5.2858	4.0839
57	290/0	7.1664	0.9526
58	300/1	35.4656	0.8932
59	290/1	10.9895	0.8653
60	300/3	115.7327	4.8387
61	300/1	26.7803	0.7141
62	290/2	9.3222	2.8809
63	290/1	16.9436	0.9469
64	300/0	27.7014	1.7794
65	290/1	55.1891	4.9226
66	310/6	620.3234	8.0999
67	400/2	23.6969	0.5595
68	290/2	36.63757	1.4766
69	290/2	241.5044	11.7279
70	290/1	5.5229	0.3386
71	290/2	173.1734	7.0318
72	300/2	77.4627	3.2468
